# Methane Carbon Supports Aquatic Food Webs to the Fish Level

**DOI:** 10.1371/journal.pone.0042723

**Published:** 2012-08-07

**Authors:** Angela M. Sanseverino, David Bastviken, Ingvar Sundh, Jana Pickova, Alex Enrich-Prast

**Affiliations:** 1 Institute of Biology, University Federal of Rio de Janeiro, Rio de Janeiro, Brazil; 2 Department of Thematic Studies–Water and Environmental Studies, Linköping University, Linköping, Sweden; 3 Uppsala BioCenter, Department of Microbiology, Swedish University of Agricultural Sciences, Uppsala, Sweden; 4 Uppsala BioCenter, Department of Food Science, Swedish University of Agricultural Sciences, Uppsala, Sweden; University of Delaware, United States of America

## Abstract

Large amounts of the greenhouse gas methane (CH_4_) are produced by anaerobic mineralization of organic matter in lakes. In spite of extensive freshwater CH_4_ emissions, most of the CH_4_ is typically oxidized by methane oxidizing bacteria (MOB) before it can reach the lake surface and be emitted to the atmosphere. In turn, it has been shown that the CH_4_-derived biomass of MOB can provide the energy and carbon for zooplankton and macroinvertebrates. In this study, we demonstrate the presence of specific fatty acids synthesized by MOB in fish tissues having low carbon stable isotope ratios. Fish species, zooplankton, macroinvertebrates and the water hyacinth *Eichhornia crassipes* were collected from a shallow lake in Brazil and analyzed for fatty acids (FA) and carbon stable isotope ratios (δ^13^C). The fatty acids 16∶1ω8c, 16∶1ω8t, 16∶1ω6c, 16∶1ω5t, 18∶1ω8c and 18∶1ω8t were used as signature for MOB. The δ^13^C ratios varied from −27.7‰ to −42.0‰ and the contribution of MOB FA ranged from 0.05% to 0.84% of total FA. Organisms with higher total content of MOB FAs presented lower δ^13^C values (i.e. they were more depleted in ^13^C), while organisms with lower content of MOB signature FAs showed higher δ^13^C values. An UPGMA cluster analysis was carried out to distinguish grouping of organisms in relation to their MOB FA contents. This combination of stable isotope and fatty acid tracers provides new evidence that assimilation of methane-derived carbon can be an important carbon source for the whole aquatic food web, up to the fish level.

## Introduction

Methane (CH_4_) is the terminal product of anaerobic respiration when all electron acceptors except carbon dioxide (NO_3_^−^, Mn^+4^, Fe^+3^ and SO_4_^−2^) have been depleted by the microbial community. The production of methane is performed by methanogens, which are microorganisms belonging to the domain Archaea.

In 1906, Sohngen [1] showed for the first time that CH_4_ can serve as an energy and carbon source for bacteria. Biological oxidation of CH_4_ is now known to occur aerobically in both terrestrial and aquatic habitats [2], and anaerobically in sediments and anoxic salt water [3]. The aerobic methane oxidizing bacteria (MOB) have been classified into the phylum Proteobacteria and recently also Verrucomicrobia (based on three strains), the latter representing thermophilic acidophiles and still being under taxonomic debate [4]. The MOB in Proteobacteria have 16 recognized genera within the classes Gammaproteobacteria (traditionally referred to as “Type I” MOB) and Alphaproteobacteria (“Type II” MOB) [5], [6], although some genera do not fit these generalizations very well [4].

Aquatic food webs are supported by organic matter derived from phytoplankton, macrophytes or imported from the surrounding land (allochthonous sources) [7], [8]. CH_4_ production accounts for a large proportion of the total organic matter degradation in freshwater lakes (20–56%) [9], and is a source of energy and carbon for aerobic MOB in the water column and the sediment, linking the anoxic and oxic communities [10]–[12].

Isotopic distributions in animals are generally closely related to dietary isotopic composition [13]. The stable isotopic compositions of animal tissues reflect both long-term and short-term diets in slow and fast turnover tissues [14]. Measurement of carbon stable isotope ratios (δ^13^C) has been a successful tool in food web studies [15]–[17]. Because CH_4_ is much depleted in ^13^C, low δ^13^C values in organisms of aquatic food webs have been considered to indicate their consumption of CH_4_-derived carbon [10], [11]. Such low values of δ^13^C in animal biomass have been reported for invertebrates in various aquatic systems [11], [15], [18]–[22].

Low δ^13^C values have been reported for fish, and a few studies have suggested that CH_4_-derived carbon could be transferred in substantial amounts to higher trophic levels [11], [23]–[26]. Calheiros ([25] unpublished data) found strongly negative δ^13^C values for zooplankton, aquatic insects and detritivorous fishes in a Brazilian Pantanal lake, being the first study to discuss the importance of methanotrophs for the whole food web in this wetland.

While more negative δ^13^C values can be an indicator of CH_4_ carbon, the isotopic signal from other potential sources of ^13^C-depleted carbon – such as primary production based on CO_2_ from respiration [27] – can provide alternative explanations [16], [17]. Therefore, the combination of stable isotope analysis with other independent biomarkers is needed to elucidate if low δ^13^C values are due to assimilation of carbon from biogenic CH_4_ [11], [28].

The lipids of the proteobacterial MOB have special fatty acid (FA) composition [5], [6], [29], [30]. They contain a few very unusual FAs that have been used as efficient group-specific markers in studies of abundance and dynamics in methanotrophic community structure [31], [32]. A fraction of the assimilated FAs of prey organisms is stored in cells rather than being degraded and the chain lengths and double bond positions in such stored FAs are preserved [33]. On a general level, variation in the FA composition using specific MOB FA can be used as a traceability tool for determining the relative dependency on bacteria versus phytoplankton in diets [34] and seasonal variation in food quality used by zooplankton [35].

Similarly, monounsaturated fatty acids, like those diagnostic for MOB, can thus be microbial biomarkers indicating transfer of carbon and energy from methane to higher food web levels. The assumption that animals actually ingest MOB has been supported by the detection of phospholipid fatty acids (PLFAs) diagnostic for MOB in tissues of chironomid larvae [36]. In addition, Deines *et al.* [18] experimentally confirmed that MOB carbon can be transferred to invertebrate animals. In this way, findings that FAs specific for MOB are present in tissues of ^13^C-depleted freshwater invertebrates have been supporting the hypothesis of a link between MOB and animal food webs [36]. However, MOB specific FAs have not been reported in fishes so far.

In this study, we tested the hypothesis that methane carbon from MOB can be transferred through food webs all the way to fish, by combining analyses of δ^13^C and fatty acid composition in fish, benthic macroinvertebrates, zooplankton, and the dominating aquatic macrophyte from a shallow tropical lake in Pantanal, Brazil. We found specific fatty acids of methane-oxidizing bacteria in tissues of aquatic invertebrates and fish, showing that methane can in fact contribute carbon to large parts of aquatic food webs and production of fish biomass.

## Results and Discussion

**Figure 1:**
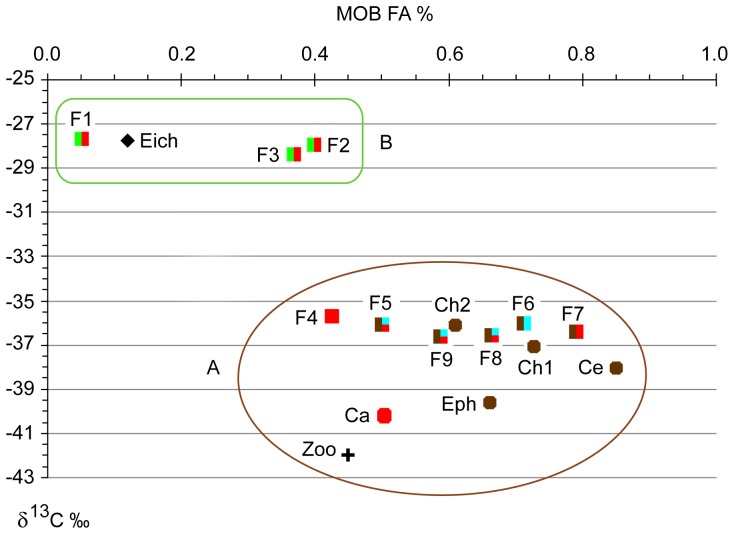
Methane oxidizing bacteria fatty acids (MOB FA %) and carbon isotope ratios (δ^13^C ‰) in organisms. MOB FA % represents the sum of the MOB fatty acid markers 16∶1ω8c, 16∶1ω8t, 16∶1ω6c, 16∶1ω5t, 18∶1ω8c and 18∶1ω8t, expressed as percentage of total fatty acids. Ca- *Chaoborus* sp., Ce- Ceratopogonidae, Ch1- Chironomidae sp.1, Ch2- Chironomidae sp.2, Eph- *Campsurus* sp., F1- *Tetragonopterus argenteus*, F2- *Astyanax* cf. *bimaculatus*, F3- *Parauchenipterus galeatus*, F4- *Crenicichla* sp., F5- *Potamorhina squamoralevis*, F6- *Cyphocharax* sp., F7- *Anadoras grypus*, F8- *Leporinus friderici*, F9- *Steindachnerina brevipinna*, Eich- *Eichhornia crassipes*, Zoo- Zooplankton. Fishes are represented by squares, macroinvertebrates by circles, zooplankton by cross and the plant by diamond. The different colors indicate the type of food sources expected to dominate: green indicates herbivory on plant material, blue herbivory on bottom filamentous algae, red carnivory and brown detritivory. Other types of feeding may occur and can result in variability. The data points separate into two major groups, denoted A and B.

All δ^13^C values ranged from −42.0 to −27.7‰ and the contribution of MOB FA varied from 0.05 to 0.84% of total FA (Figure 1). Based on previous studies the FAs 16∶1ω8c, 16∶1ω8t, 16∶1ω6c, 16∶1ω5t, 18∶1ω8c and 18∶1ω8t are signature FAs for MOB [29], [30]. The total contribution of these FAs was higher in organisms with low δ^13^C (Table 1, Figure 1). Two discrete groups arose in Figure 1: (A) organisms with a higher total content of MOB signature FAs and lower δ^13^C values, and (B) organisms with lower content of MOB signature FAs and higher δ^13^C values. The former group includes Ceratopogonidae and the fish *Anadoras grypus* with the highest contribution of MOB fatty acids (0.84 and 0.79%, respectively), Chironomidae sp.1 (0.73%), *Cyphocharax* sp. (0.71%) and the ephemeropteran *Campsurus* sp. (0.66%). These five organisms were also depleted in ^13^C (δ^13^C of −38.1, −36.5, −37.1, −36.1 and −39.7‰, respectively). All these organisms can feed at oxic-anoxic interfaces where MOB abundance is likely to be high [10], which provides an explanation to the comparatively high consumption and incorporation of MOB carbon into their biomass. Zooplankton and *Chaoborus* sp. showed the lowest δ^13^C values (−42.0 and 40.2‰, respectively) and a MOB-specific FA contribution of 0.45 and 0.50%, respectively. Calheiros ([25] unpublished data) also found low values of δ^13^C in zooplankton (−42.7 to −31.6‰), benthic chironomids (−62 to −49‰) and ephemeropterans (−41.4 to −34.3‰) in another Pantanal lake, suggesting an assimilation of biogenic CH_4_. Other taxa in group A include the fishes *Potamorhina squamoralevis*, *Crenicichla* sp., *Leporinus friderici* and *Steindachnerina brevipinna* which had equally low δ^13^C values (δ^13^C<−36‰) and a range of MOB-specific FA content from 0.43 to 0.66%. These fishes can all have benthivorous and omnivorous or detritivorous feeding habits, except *Crenicichla* sp., which is mainly carnivorous.

**Table 1:** Isotope ratios of carbon and contribution of the methane oxidizing bacteria fatty acid markers.

Organisms	δ^13^C	16∶1ω8c	16∶1ω8t	16∶1ω6c	16∶1ω5t	18∶1ω8c	18∶1ω8t	MOB FA %
*Eichhornia crassipes*	−27.8	0.0546	0.00098	0.00977	0.00559	0.043	0.0081	0.12
Invertebrates								
Chironomidae sp.1	−37.1	0.242	0	0.0862	0.153	0.23	0.0175	0.73
Chironomidae sp.2	−36.2	0.209	0.00886	0.0533	0.0938	0.236	0.0102	0.61
*Chaoborus* sp.	−40.2	0.0432	0	0.0199	0.0146	0.428	0	0.50
Ceratopogonidae	−38.1	0.32	0.02	0.0856	0.17	0.237	0.0094	0.84
*Campsurus* sp.	−39.7	0.0697	0.00881	0.0648	0.268	0.248	0	0.66
Zooplankton	−42.0	0.118	0	0.0102	0.0106	0.308	0	0.45
“Fishes”								
*Tetragonopterus argenteus*	−27.7	0.00565	0	0.00223	0.00763	0.0239	0.0074	0.05
*Astyanax cf.bimaculatus*	−28.0	0.184	0	0.0272	0.0804	0.106	0	0.40
*Parauchenipterus galeatus*	−28.4	0.155	0	0.0344	0.103	0.0742	0	0.37
*Crenicichla* sp.	−35.8	0.179	0	0.0363	0.105	0.113	0	0.43
*Potamorhina squamoralevis*	−36.1	0.177	0	0.0596	0.0903	0.174	0	0.50
*Cyphocharax* sp.	−36.1	0.304	0	0.0906	0.147	0.168	0	0.71
*Anadoras grypus*	−36.5	0.183	0	0.0682	0.0914	0.45	0	0.79
*Leporinus friderici*	−36.6	0.28	0	0.0801	0.222	0.0817	0	0.66
*Steindachnerina brevipinna*	−36.7	0.238	0	0.0988	0.145	0.105	0	0.59

δ^13^C, carbon stable isotope ratios relative to the PeeDee Belemnite standard and expressed as a per mil (‰) deviation; MOB FA, methane oxidizing bacteria fatty acid markers 16∶1ω8c, 16∶1ω8t, 16∶1ω6c, 16∶1ω5t, 18∶1ω8c and 18∶1ω8t, expressed as percentage of total fatty acids. MOB FA % represents the sum of the MOB FA markers.

A higher variability in FA composition than in δ^13^C could be explained by physiological differences among taxa regarding to what extent various FAs are metabolized or stored in muscle tissue. Branched FA are often difficult to metabolize and can therefore be magnified in the tissues. Lipid composition in fish muscle varies mainly with fish diet. The metabolism of total lipids and fatty acids might be related to age, sex, reproductive cycle and capture period of the fish and influenced by environmental factors as seasonal hydrological cycle and food availability [37]. Besides, lipids are unevenly stored among tissue types throughout the year. Differences in migratory behavior and life history may explain distinctive lipid dynamics among fish species [38]. In addition, some organisms might have a more homogeneous diet which could lead to a lower isotopic variation. Calheiros ([25] unpublished data) reported low δ^13^C values for *P. squamoralevis* (mean −36.9‰) and small variations in isotopic signals among seasons, which could be due to a more specialized diet. Wantzen [24] pointed out that seasonal variations in isotopic signal of fish species in Pantanal were more prominent in less specialized omnivores, invertivores and some carnivores, while more specialized detritivores and herbivores appeared to be more influenced by δ^13^C changes in the diet affected by biogeochemical processes.

Group B (Figure 1), formed by the less ^13^C-depleted fish species *Tetragonopterus argenteus* (−27.7‰), *Astyanax cf. bimaculatus* (−28.0‰) and *Parauchenipterus galeatus* (−28.4‰), had lower contents of MOB-specific FA (0.05, 0.40 and 0.37%, respectively). The higher δ^13^C values in combination with lower MOB FA content of these three fishes indicate a lower incorporation of MOB biomass. This conclusion is in accordance with their feeding habits. These taxa are known to be pelagic or living close to aquatic plants, presenting omnivorous habits with tendency to herbivory-invertivory [39], [40]. Wantzen et al. [24] found similar isotopic signatures for *T. argenteus* and *A. bimaculatus* in Pantanal. Although aquatic C_4_ plants were not the major carbon source, they suggested that at least part of the *A. bimaculatus* diet would be derived from aquatic C_4_ grasses, which have higher δ^13^C values than C_3_ plants and occur in small patches among large mats of C_3_ macrophytes [24]. The water hyacinth *Eichhornia crassipes*, a C_3_ plant that totally dominates the macrophyte community in the studied environment, had a similar δ^13^C signature (−27.8‰). Therefore, a combination of carbon sources from C_3_ plants, C_4_ plants and MOB could yield a ^13^C signature similar to C_3_ plants.

Traces of MOB FA found in plant samples might have two different causes. First, MOB FA in plant samples could be due to the difficulty to completely remove biofilms from the plant surface, especially those attached to the submerged roots. Secondly, there are studies that revealed the presence of MOB inside living tissues of submerged aquatic plants (*Sphagnum* mosses) [41], [42]. Methane-derived carbon was incorporated into plant lipids when mosses were submerged [42]. Even though *E. crassipes* is a floating macrophyte and not a moss, we cannot exclude the possibility of this phenomenon.

**Figure 2:**
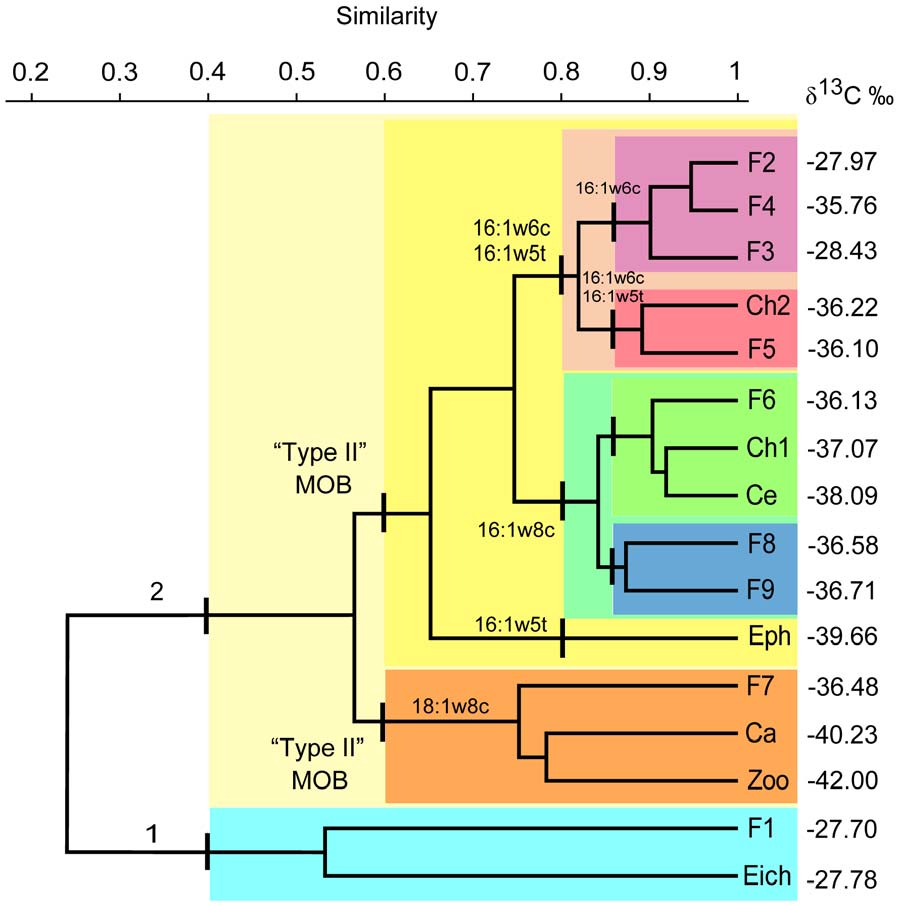
Dendrogram from UPGMA cluster analysis based on a Bray-Curtis similarity matrix of organisms according to MOB FA contributions. The isotope ratios of carbon (δ^13^C ‰ vs. PDB) were included for comparison. Ca- *Chaoborus* sp., Ce- Ceratopogonidae, Ch1- Chironomidae sp.1, Ch2- Chironomidae sp.2, Eph- *Campsurus* sp., F1- *Tetragonopterus argenteus*, F2- *Astyanax* cf. *bimaculatus*, F3- *Parauchenipterus galeatus*, F4- *Crenicichla* sp., F5- *Potamorhina squamoralevis*, F6- *Cyphocharax* sp., F7- *Anadoras grypus*, F8- *Leporinus friderici*, F9- *Steindachnerina brevipinna*, Eich- *Eichhornia crassipes*, Zoo- Zooplankton. The FA 16∶1ω8c, 16∶1ω8t, 16∶1ω6c, 16∶1ω5t, 18∶1ω8c and 18∶1ω8t were used as MOB markers.

The dendrogram in Figure 2 illustrates the clustering of organisms based on contributions of the different MOB FAs only (i.e. with no consideration to ^13^C signatures). In contrast to the grouping based on total MOB FA and ^13^C values illustrated in Figure 1, this clustering approach depends on the relative contribution of the individual MOB FAs, but yields a similar overall result. Four arbitrary “cutoff” lines, at Bray-Curtis similarities of 0.4, 0.6, 0.8 and 0.87 were used as reference points for identifying clusters.

At a similarity of 0.4, two distinct clusters can be seen. Cluster *1* grouped organisms with the lowest contributions of MOB FA, i.e. *E. crassipes* and *T. argenteus*. This is in accordance with the fact that *T. argenteus* is an omnivorous fish with tendency to herbivory-insectivory, feeding mainly on vascular plants [43], plant debris and terrestrial insects [39], [44]. Cluster *2* was formed by all other organisms with higher MOB FA values.

At a similarity of 0.6, cluster *2* was divided into two new clusters: One had a high contribution of the FA 18∶1ω8c, which is highly specific for type II MOB [5], and was therefore named *Type II MOB*. The other cluster was based on high contribution of specific FA markers for MOB type I, and named *Type I MOB*. Inside cluster *Type II MOB*, *Chaoborus* sp. and zooplankton were grouped together since they presented the highest contributions of 18∶1ω8c (84.6% and 68.9%, of the total MOB FA, respectively), followed by *A. grypus* (56.7% of the total MOB FA). Chaoborid larvae are zooplankton predators, whose preferred food source are microcrustaceans, although they eat a wide variety of animals including dipteran larvae, oligochaetes, rotifers, other chaoborids [45] and even dinoflagellates [46]. It should be noted that zooplankton in this shallow tropical lake are not restricted to autotrophic or heterotrophic production in the water column, but can also access food items from surface sediments which are frequently resuspended, and from the biofilms in the root zone of floating *E. crassipes. Anadoras grypus* is an invertivore, bottom-feeding fish [47].

Within cluster *Type I MOB*, three clusters were identified at a “cutoff” similarity of 0.8:

- The ephemeropteran *Campsurus* sp. which showed the highest content of the FA 16∶1ω5t. Nymphs of *Campsurus* are bottom collectors/gatherers [48] and construct their tunnels in soft substrates like mud or sand [49].
- A cluster of organisms with the highest contributions of 16∶1ω8c which are mainly benthivorous and iliophagous (“mud eaters”; *Cyphocharax* sp., Chironomidae sp.1, Ceratopogonidae, *L. friderici* and *S. brevipinna*). Inside this cluster, two sub-groups emerged: one formed by *Cyphocharax* sp., Ceratopogonidae and Chironomidae sp.1, and another with *L. friderici* and *S. brevipinna*. Species of *Cyphocharax* pick mainly biofilms from the bottom substrates [50]. Larvae of the aquatic insects Ceratopogonidae and Chironomidae rely on different food sources. Some are detritivorous while others may feed also on periphyton [51]. Both families have collector and scraper feeding habits, live close to the bottom and/or associated to roots of aquatic plants [48]. The last two species *L. friderici* and *S. brevipinna* were the most ^13^C depleted fishes and they have similar feeding habits. Both fishes are iliophagous and feed on detritus, benthic filamentous algae and invertebrates associated to bottom substrates [52], [53].
- Two groups with high contributions of 16∶1ω6c and 16∶1ω5t (*A.* cf. *bimaculatus*, *Crenicichla* sp., *P. galeatus*, Chironomidae sp.2 and *P. squamoralevis*). The group of Chironomidae sp.2 and *P. squamoralevis* showed similar proportions of both fatty acids 16∶1ω6c and 16∶1ω5t, while *A.* cf. *bimaculatus*, *Crenicichla* sp. and *P. galeatus* were similar to each other in terms of primarily 16∶1ω6c. *Potamorhina squamoralevis* feeds mainly on detritus and benthic algae and invertebrates associated with bottom sediments [54]. *Astyanax bimaculatus* is an omnivorous fish which consumes mainly insects [55]. Species of *Crenicichla* are stalking predators, feeding on insects and fishes [56]. The diet of *P. galeatus* consists mainly of terrestrial and aquatic insects, but also aquatic invertebrates, fishes, fungi, algae, higher plants and detritus [40].

**Figure 3:**
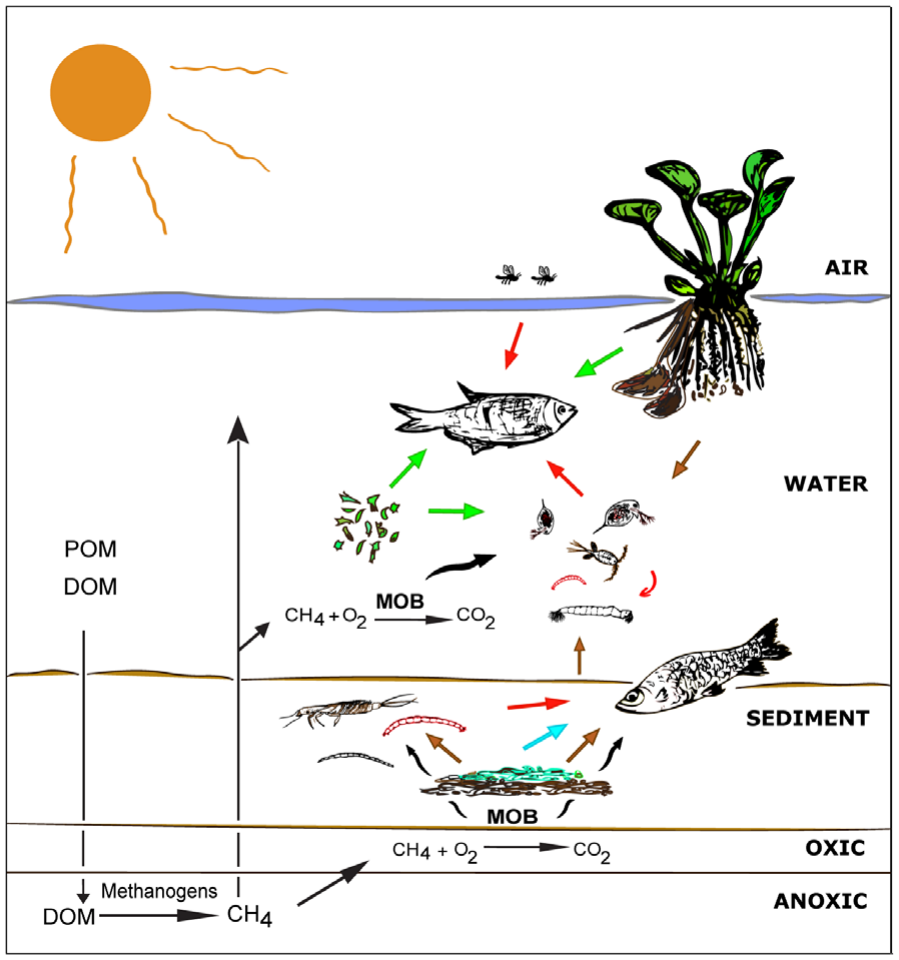
Schematic illustration of the incorporation of carbon of aerobic methane oxidizing bacteria (MOB) into the food web of a tropical shallow lake. Particulate and dissolved organic matter (POM and DOM, respectively) of autochthonous and allochthonous origin support anaerobic methane (CH_4_) formation. CH_4_ is oxidized by MOB and CH_4_-derived carbon is transferred to higher trophic levels subsidizing pelagic and benthic organisms, reaching the fish level. Arrows with different colors indicate potential food sources: black- MOB; green- herbivory on phytoplankton and/or plant material; blue- herbivory on bottom filamentous algae; red- carnivory on aquatic and/or terrestrial organisms; brown- detritivory.

The presence of MOB signature FAs in benthic aquatic insects, planktonic organisms as chaoborids and zooplankton, and fishes showed the importance of biogenic methane to different aquatic compartments in this Pantanal ecosystem (Figure 3). We used a simple two-source mixing model based on ^13^C signatures to estimate the relative importance of methane-derived carbon as described by Trimmer et al. [22]. Assuming *E. crassipes* and MOB as end members, it was estimated that methane oxidation contributes on average 13% and 1% to the carbon content in fishes from group A and B respectively (Figure 1). However, this value is only an estimate, as the ^13^C signature of many important carbon sources (phytoplankton, terrestrial DOM from C_3_ and C_4_ plants) were not determined and could not be included in this model. The flood pulse also changes the lake dynamics, and may affect the relative importance of MOB for the food chain. We applied the same model for aquatic insects and zooplankton from this lake and obtained a contribution of 16% and 22% as carbon source respectively, a similar value as the results reported by Ravinet et al. [23] and Trimmer et al. [22].

In the present study, the relationship between contribution of MOB FAs and δ^13^C seems to be driven more by the MOB type II FA (18C) than by MOB Type I FAs (16C). It is possible that MOB type II dominates over type I in this wetland and the Pantanal floodplain in general. Sundh et al. [31] pointed out that most studies in temperate and boreal lakes show a predominance of type I over type II, while a few studies reported dominance of MOB type II in tropical freshwaters (e.g. [57], [58]). Along the same lines, a recent study indicated that type II MOB might drive CH_4_ oxidation in flood-pulsed wetlands [59].

The two groups of MOB have different growth characteristics and respond differently to variation in temperature, pH and concentrations of oxygen and methane. Low temperatures seem to favor the development MOB type I over type II [60]. With respect to oxygen and methane supply, MOB type II often dominates at relatively high methane and low oxygen concentrations, while MOB type I dominates at low CH_4_ and high oxygen concentrations [61].

The Pantanal floodplain is characterized by conditions that together favor production and emissions of CH_4_, such as high primary productivity, shallow depth of inundation, and high temperature [62]. Such conditions could be expected to be favorable for aerobic methane oxidizers with a probable selection for type II, in line with our results. However, although FAs of MOB type II showed a stronger correlation with ^13^C signatures, we have to be careful to draw the conclusion that MOB type II dominates fish consumption of MOB in Pantanal as the actual levels of MOB type I and type II FAs in the fishes were overall quite similar.

This study showed, by combining two independent tracers (MOB FA and δ^13^C), that MOB carbon was transferred through the food web up to the fish level. Hence, our data demonstrate that CH_4_ can be a significant carbon source not only for the microbial food web and invertebrates, but also for higher trophic levels. The Pantanal region is characterized by high CH_4_ production [63] and emissions [62], and transfer of CH_4_ carbon throughout the food web to various fish species could be a common phenomenon in CH_4_-rich ecosystems.

## Materials and Methods

The Pantanal ecosystem is situated in the Northern part of the Paraguay River basin and is the largest freshwater wetland in South America. The Pantanal wetland forms a gradient from areas flooded by rainwater to areas flooded by river water [64]. The strong fluctuations between dry and flood periods throughout the year markedly affect aquatic and terrestrial communities of micro- and macro-organisms, primary and secondary production as well as nutrient dynamics. The Pantanal region is a very important environment in terms of global methane fluxes because the emissions from their aquatic systems are among the highest rates measured in tropical areas [62].

Different species of fishes, zooplankton, aquatic invertebrates and the aquatic macrophyte *Eichhornia crassipes* were collected from a shallow lake (average depth 1.8 m) in the Paraguay River flood plain near Ladário, Mato Grosso do Sul, Brazil. The fishes were provided by local inhabitants using hand-thrown nets. The captured fishes were killed instantaneously to minimize suffering and we were allowed to document the fish taxonomy and take subsamples immediately after fishing, before further use of the fish by the locals. A piece of fish dorsal muscle was cut out and freeze dried. Zooplankton were collected with a zooplankton net (150 µm mesh), rinsed thoroughly with deionized water, handpicked into 2 ml sterile Eppendorf tubes, and freeze dried. *Eichhornia crassipes* was the totally dominating macrophyte in and around the lake and whole plants including roots were sampled (this plant is floating and roots are detached from the sediment). The plants were rinsed in water and biofilms were removed from their surfaces. Sediment was collected by diving and sieved for sampling of benthic invertebrates. All benthic invertebrates were kept in filtered (Whatman GF/F) lake water for 24 hours for gut clearance, and were then rinsed and freeze dried.

Samples for stable isotope analysis were analyzed with a Carlo Erba NC2500 analyzer connected to a Finnigan MAT Delta plus mass spectrometer. Carbon stable isotope ratios were reported relative to the PDB (PeeDee Belemnite) standard and expressed as a per mil (‰) deviation according to the equation δ^13^C = {(δ^13^C/δ^12^C)_sample_/|(δ^13^C/δ^12^C)_standard_−1|}×1000. Samples with higher isotope values are relatively enriched in the heavy isotope ^13^C while samples with lower isotope values are relatively depleted in ^13^C and enriched in the light isotope ^12^C.

Lipids from biological samples were extracted following the method of Hara and Radin [65]. For GC analysis of fatty acids, the total lipids were methylated according to Appelqvist [66] and the FAMEs (fatty acid methyl ester) were analysed on a Varian CP-3800 Gas Chromatograph system (Agilent Technologies, Santa Clara, CA, USA) using the same column and conditions as in Trattner et al. [67]. Fatty acid peaks were identified by comparison with retention times obtained for the standard fatty acid mixture GLC standard 461 (Nu-Chek Prep, Elysian, MN, USA). Peak area integration was performed using Star chromatography workstation software version 5.5 (Varian AB, Stockholm, Sweden). The relative contributions of the different double bond positions and isomers of the monounsaturated FAs were determined after DMDS derivatization and GC-MS analyses as described previously [68], [69]. These data were then used to split the total amounts of monounsaturated FAs obtained in the GC-FID analyses of the FAMEs into the different double-bond positions and isomers. The fatty acids 16∶1ω8c, 16∶1ω8t, 16∶1ω6c, 16∶1ω5t, 18∶1ω8c and 18∶1ω8t were considered markers for MOB [5], [29], [30].

A cluster analysis was performed to recognize grouping of organisms according to their contents of the different MOB FA markers 16∶1ω8c, 16∶1ω8t, 16∶1ω6c, 16∶1ω5t, 18∶1ω8c and 18∶1ω8t (% of total FA) and to identify differences among those groups. The hierarchical clustering was carried out on a Bray-Curtis similarity matrix (organisms as objects and MOB FA as descriptors) and represented by a dendrogram produced by UPGMA (Unweighted Pair-Group Method with Arithmetic averages) clustering of this resultant matrix. The cluster analysis was performed using the software package PAST version 2.12 [70].

In order to make an approximation of the relative contribution of CH_4_-derived carbon for fish, macroinvertebrates and zooplankton, a simple two-source mixing model was used (x = {c−b}/{a−b}) as suggested by Trimmer et al. [22]. We assumed *E. crassipes* (a) and MOB (b) as dietary end members. The δ^13^C values of fish, zooplankton and aquatic insects represented the mixture (c). Trophic fractionation was assumed to be negligible. The δ^13^C values of MOB were derived from methane δ^13^C values measured for the lake (mean: −80.9‰) by Conrad et al. [63] with a further fractionation by the MOB of 0–16% to give a potential range for MOB of −96.9 to −80.9‰ [22], [71].
