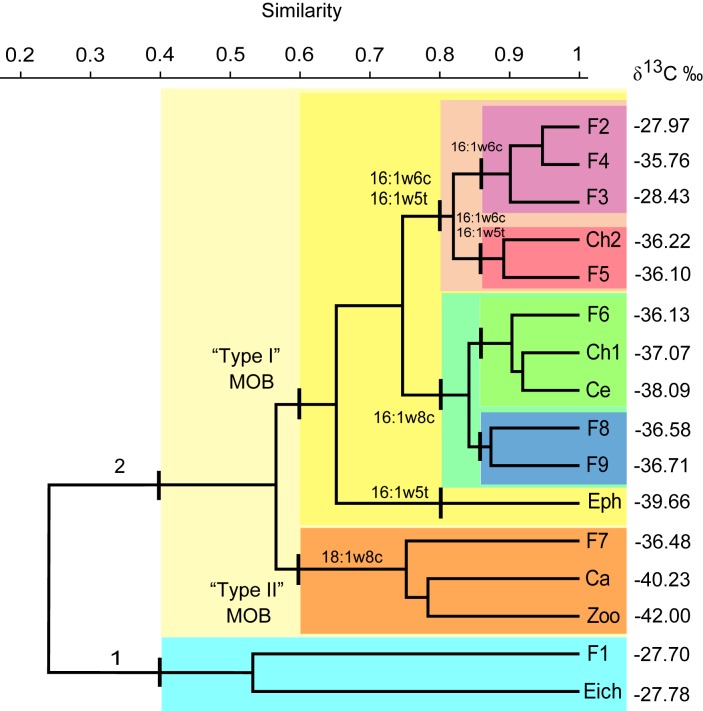# Correction: Methane Carbon Supports Aquatic Food Webs to the Fish Level

**DOI:** 10.1371/annotation/a1ff8f9e-ebdc-479d-96d3-d01ecdf26b9e

**Published:** 2013-01-17

**Authors:** Angela M. Sanseverino, David Bastviken, Ingvar Sundh, Jana Pickova, Alex Enrich-Prast

There was an error in the text in Figure 2. "Type II" MOB was incorrectly used twice. Please see the corrected Figure 2 here: 

**Figure:**